# A comparative mutation–selection reanalysis of colon and rectal adenocarcinoma highlights PIK3CA variants and BRAF V600E

**DOI:** 10.1186/s12967-026-08754-2

**Published:** 2026-08-05

**Authors:** Liangze Ke, Michael Cecchini, Jeffrey P. Townsend

**Affiliations:** 1 Department of Biostatistics, Yale School of Public Health, New Haven, CT USA; 2 Program in Developmental Therapeutics, Yale Cancer Center, New Haven, CT USA; 3 Department of Medicine, Yale School of Medicine, New Haven, CT USA; 4 Program in Genetics, Genomics, and Epigenetics, Yale Cancer Center, New Haven, CT USA; 5 Department of Ecology and Evolutionary Biology, Yale University, New Haven, CT USA

A recent multi-omic comparison of colon adenocarcinoma (COAD) and rectal adenocarcinoma (READ) by De Angelis et al. [1] provided evidence that these anatomically adjacent malignancies are biologically distinct diseases. Their interpretation drew on higher copy-number alteration burdens in COAD, largely non-overlapping sets of differentially expressed genes with distinct pathway enrichment and clinical associations, and differences in mutational spectra as well as inferred driver compositions. On this basis, the authors recommended separating colorectal cancer into colon and rectum analytically and clinically, and highlighted six putatively “private” drivers prioritized through prevalence- and network-based criteria. However, this association-based prioritization does not quantify the key question: whether specific mutations confer differential selective advantage in their tissue context, which prevalence differences alone cannot establish. Biologically meaningful distinctions would manifest as differences in growth and proliferation quantified by estimates of selection strength accounting for background mutation rates [2]. We therefore quantified mutation rates and selection strength on somatic variants in COAD and READ.

To evaluate this, we used trinucleotide mutational signature decomposition (MutationalPatterns [3]; COSMIC v3.4 [4]). Both tumor types were dominated by endogenous, clock-like mutational processes [5]. COSMIC SBS 1 accounted for *∼*40% of mutations in COAD (39.7%) and READ (42.1%). COAD showed relative enrichment of MMR-associated SBS6 (5.8% vs. 0.5%), SBS15 (4.3% vs. 1.8%), and SBS20 (2.3% vs. 0.1%), whereas READ showed modestly higher contributions from clock-like processes SBS5 (+3.0%), SBS40a (+2.2%), and SBS1 (+2.3%). These adjacent tissues thus differ in mutational source composition, including dysfunctional MMR in a subset of COAD. Because MMR-associated processes generate substitutions distributed across both CpG and non-CpG trinucleotide contexts—unlike the CpG-concentrated SBS1—this composition asymmetry produces variant-specific prevalence differences independent of selection, motivating explicit estimation of selective effects.

Accordingly, we quantified strength of somatic selection (average increases in cancer cell proliferation and survival [2]), aggregating variants within genes for our initial inference. As expected given elevated MMR activity, COAD exhibited a higher average mutation burden than READ (0.51/megabase vs. 0.28/megabase), which inflates the per-patient baseline mutation rate denominator in *γ* estimation and induces a cohort-level multiplicative scale offset independent of selection. We therefore aligned COAD ($\gamma_C$) and READ ($\gamma_R$) on a common scale using a global log_10_ shift ($\Delta=\operatorname{median}\!\left[\log_{10}\!\left(\gamma_{\mathrm{R}}\right)-\log_{10}\!\left(\gamma_{\mathrm{C}}\right)\right]$), and rescaling READ by $10^{\Delta}$ (here, *Δ = 1.09*).

After scaling, gene-wide selection coefficients are largely concordant between COAD and READ, with most genes exhibiting comparable effects (Fig. 1 and 2). Notably, the six “private” genes named by De Angelis et al. [1] (COAD: *ACVR1B*, *LTBP4*, *SETD1A*; READ: *C4BPA*, *EHD1*, *PPP3CC*) are at parity, exhibiting moderate selection ($10^{2}$–$10^{3}$) and only minor COAD–READ differences, and are not among the strongest compound effects in either cohort. In contrast, the upper tail of the gene-wide distribution instead comprised genes not included by De Angelis et al. Several contrasts in gene-mutation prevalence that are examined by De Angelis et al. [1]—in *PIK3CA*, *TP53*, *NRAS*, and *BRAF*—do not translate to substantial differences in aligned selection at a gene-wide scale. This lack of selective difference provides insight into how mutation prevalence reflects both mutational supply and selection, whereas meaningful distinctions in cellular and tissue function are captured by selection. Indeed, the selective architecture between COAD and READ does not support treating the nominated “private” genes as cancer-type-specific selective drivers.

**Fig. 1:**
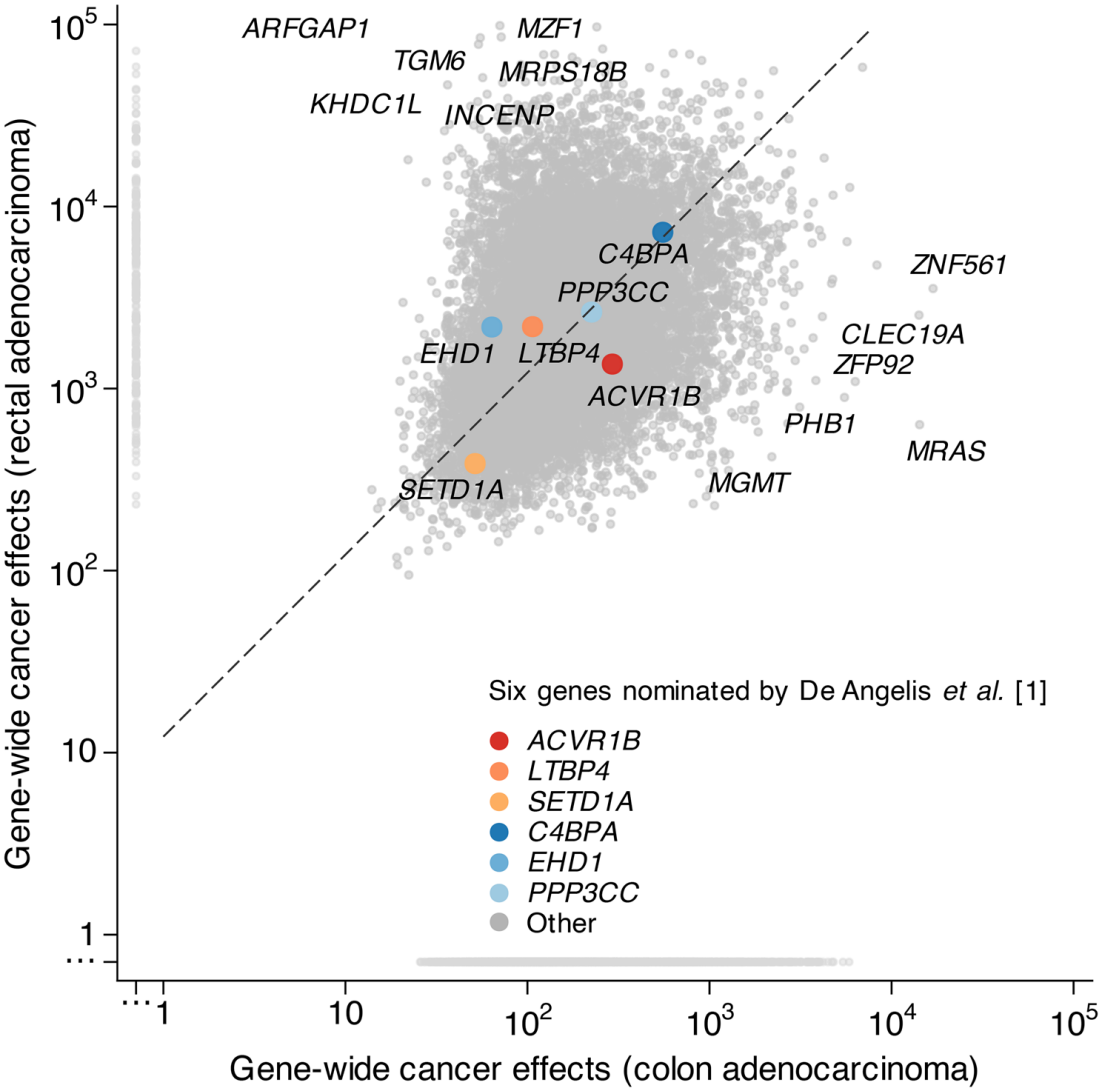
Gene-wide cancer effects in colon and rectal adenocarcinoma. Gene-level cancer effect estimates (grey circles) aggregate selection across all observed variants within a gene, including those nominated by by De Angelis et al. [1] (colored circles); and shift-aligned parity (dashed line) after correcting the global colon and rectal adenocarcinoma scale difference

**Fig. 2:**
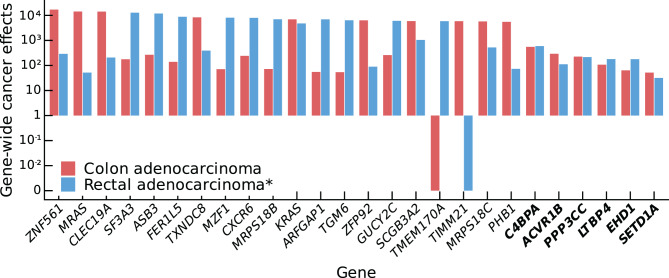
Top 10 gene-wide cancer effects in colon and rectal adenocarcinoma plus cancer effects for the six genes previously identified as “private”. Genes are sorted by the cross-cohort maximum, and those identified by De Angelis et al. [1] are in boldface. *: Rectal adenocarcinoma effects are shift-aligned to the colon adenocarcinoma cohort scale

A key limitation of all gene-wide analyses, including gene-wide *γ*, is that they shoehorn heterogeneous variants within a gene into a single estimate. Such an aggregation is sensitive to gene length, obscures functional heterogeneity by conflating high-impact hotspots with dispersed low-impact variants, and assumes co-estimable effects despite well-established position- and domain-specific mechanisms. Because selection operates on individual variants, variant-level selective advantages more directly quantify the cancer effects of specific substitutions and provide resolution better aligned with actionable biology. Analyzing selection at the variant level after scale alignment (*Δ = 1.03*) identifies a combination of high selection strength with a pronounced COAD–READ difference in PIK3CA H1047R, E545K, and especially, BRAF V600E, each exhibiting a substantially greater selective advantage in COAD than in READ (Fig. 3). By contrast, canonical KRAS hotspots and truncating APC variants show only minor differences. Thus, the principal interpretable divergences between COAD and READ localize to a small set of actionable variants implicating BRAF/MAPK- and PI3K-pathway biology.

**Fig. 3:**
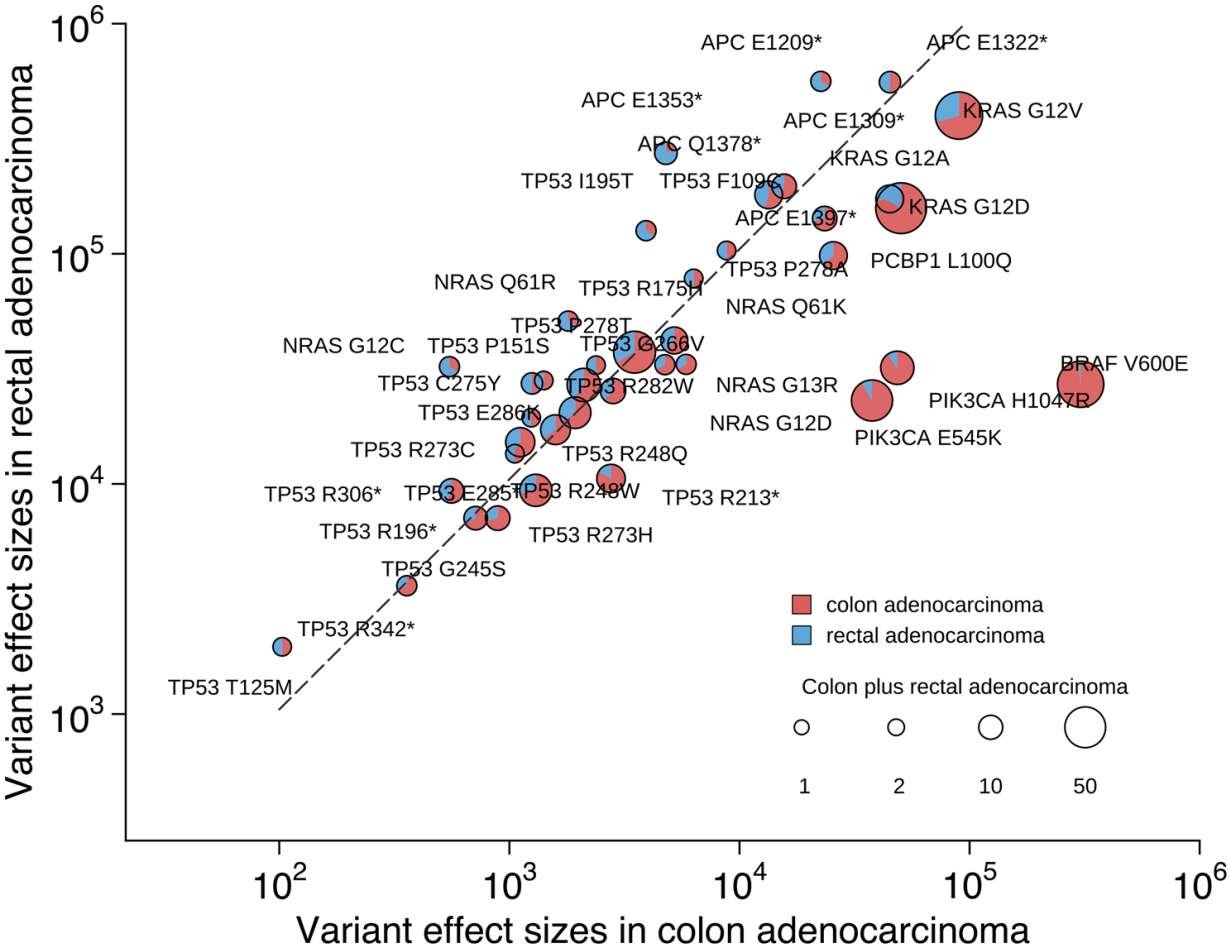
Strengths of somatic selection and prevalences of variants in colon adenocarcinoma and rectal adenocarcinoma. Strengths of somatic selection (bubbles) are shift-aligned (dashed line: shift-aligned parity). Cross-cohort numbers (bubble areas) of colon adenocarcinomas (red) and rectal adenocarcinoms (blue) harboring the variant are split by relative prevalence

BRAF-targeted therapy with encorafenib in combination with cetuximab is established for metastatic colorectal cancer with BRAF V600E mutation, while targeting PI3K remains investigational. Currently, clinical trials frequently stratify outcomes by left- versus right-sided tumors but rarely by colon versus rectum for these targeted therapies. Thus, the clinical evidence base is evolving and does not yet match the anatomical specificity of variant-level selection. We therefore identify primary-site stratification as a clear evidence gap and—aligned with De Angelis et al. [1]—encourage future trials and pooled analyses to pre-specify and report COAD–READ and other anatomical divisions alongside molecular stratification by BRAF and PIK3CA hotspot status.

## Data Availability

The dataset analysed during the current study are available in the TCGA Research Network: https://www.cancer.gov/tcga.

## Abbreviations

COAD: Colon adenocarcinoma
READ: Rectal adenocarcinoma
MMR: Mismatch repair
SBS: Single-base substitution
TCGA: The Cancer Genome Atlas

## References

[1] De Angelis MT, Rizzuto A, Amaddeo A, Sagnelli C, Vono N, Reda M, et al. Distinctive chromosomal, mutational and transcriptional profiling in colon versus rectal cancers. J Transl Med. 2025;23(1):869. 10.1186/s12967-025-06908-2. 40770356 10.1186/s12967-025-06908-2 PMC12330160

[2] Mandell JD, Cannataro VL, Townsend JP. Estimation of neutral mutation rates and quantification of somatic variant selection using cancereffectsizeR. Cancer Res. 2023;83(4):500–05. 10.1158/0008-5472.CAN-22-1508. 36469362 10.1158/0008-5472.CAN-22-1508 PMC9929515

[3] Blokzijl F, Janssen R, van Boxtel R, Cuppen E. MutationalPatterns: comprehensive genome-wide analysis of mutational processes. Genome Med. 2018;10(1):33. 10.1186/s13073-018-0539-0. 29695279 10.1186/s13073-018-0539-0 PMC5922316

[4] Alexandrov LB, Kim J, Haradhvala NJ, Huang MN, Tian Ng AW, Wu Y, et al. The repertoire of mutational signatures in human cancer. Nature. 2020;578(7793):94–101. 10.1038/s41586-020-1943-3. 32025018 10.1038/s41586-020-1943-3 PMC7054213

[5] Cannataro VL, Mandell JD, Townsend JP. Attribution of cancer origins to endogenous, exogenous, and preventable mutational processes. Mol Biol Evol. 2022;39(5):msac084. 10.1093/molbev/msac084. 10.1093/molbev/msac084 PMC9113445 35580068

